# Supplementary material for: SRSF1-dependent nuclear export inhibition of C9ORF72 repeat transcripts prevents neurodegeneration and associated motor deficits
Source: Nat Commun. 2017 Jul 5;8:16063. doi: 10.1038/ncomms16063 (PMC5504286; doi:10.1038/ncomms16063)
Supplement: Supplementary Information [file ncomms16063-s1.pdf]

File name: Supplementary Information
Description: Supplementary Figures, Supplementary Table and Supplementary Notes

File name: Peer Review File
Description:

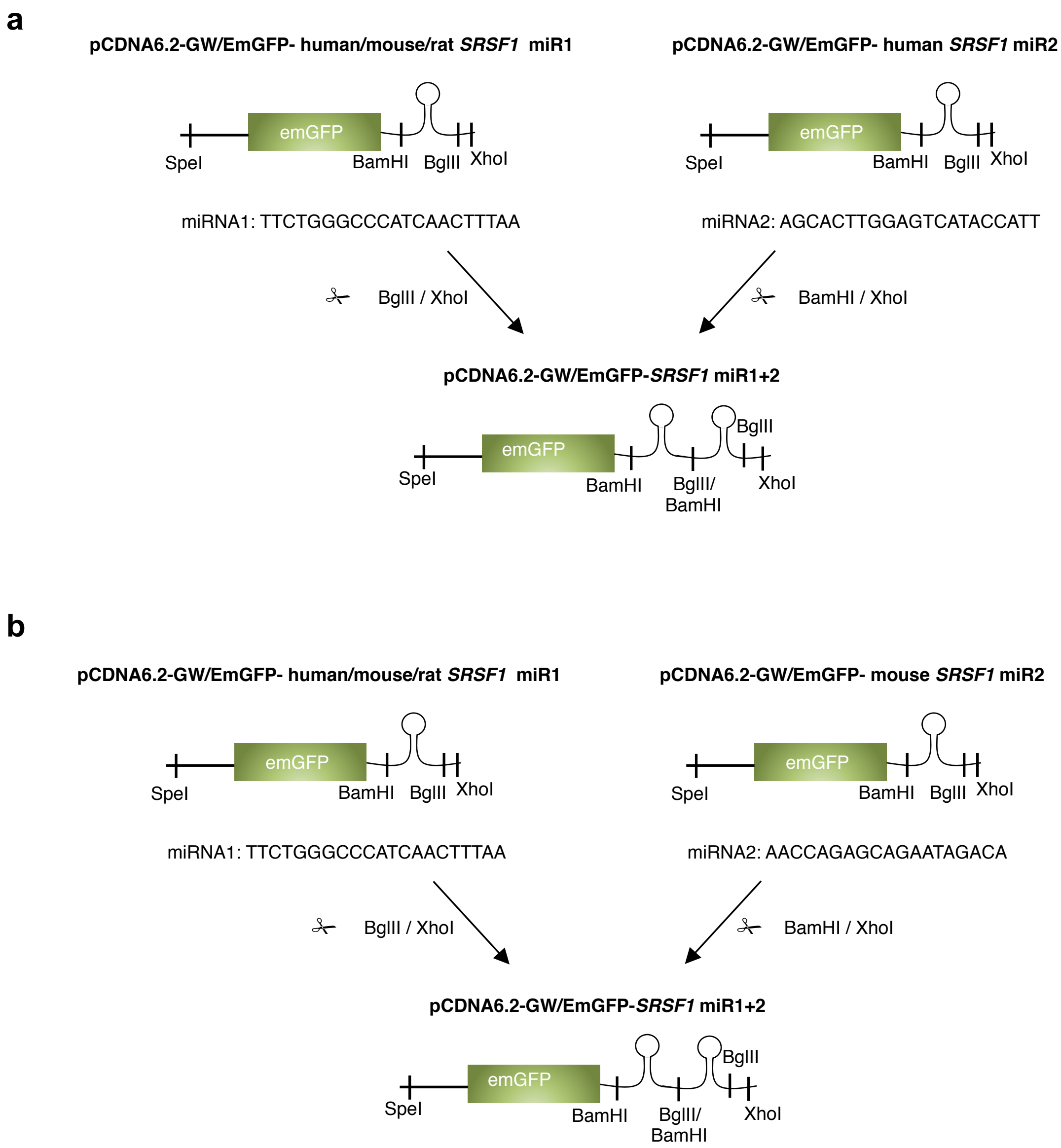

Supplementary Figure 1 | Engineering *SRSF1*-miRNA depletion vectors. (a) Diagrams of pCDNA6.2-GW/EmGFP-*SRSF1* human//mouse/rat miR1, EmGFP-*SRSF1* human miR2 and EmGFP-*SRSF1* chained miR1+2. **(b)** Diagrams of pCDNA6.2-GW/EmGFP-*SRSF1* human//mouse/rat miR1, EmGFP-*SRSF1* mouse miR2 and EmGFP-*SRSF1* chained miR1+2. For cloning, the pCDNA6.2-GW/EmGFP-*SRSF1* miR1 and EmGFP-*SRSF1* miR2 were built separately using the BLOCK-iT Pol II miR RNAi Expression Vector Kit with EmGFP (see online methods). The *SRSF1* pre-miR2 RNAi cassette was then chained by subcloning the BamHI/XhoI-cut fragment into the BglII and XhoI sites of pcDNA6.2 GW/EmGFP-*SRSF1* miR1.

a

b

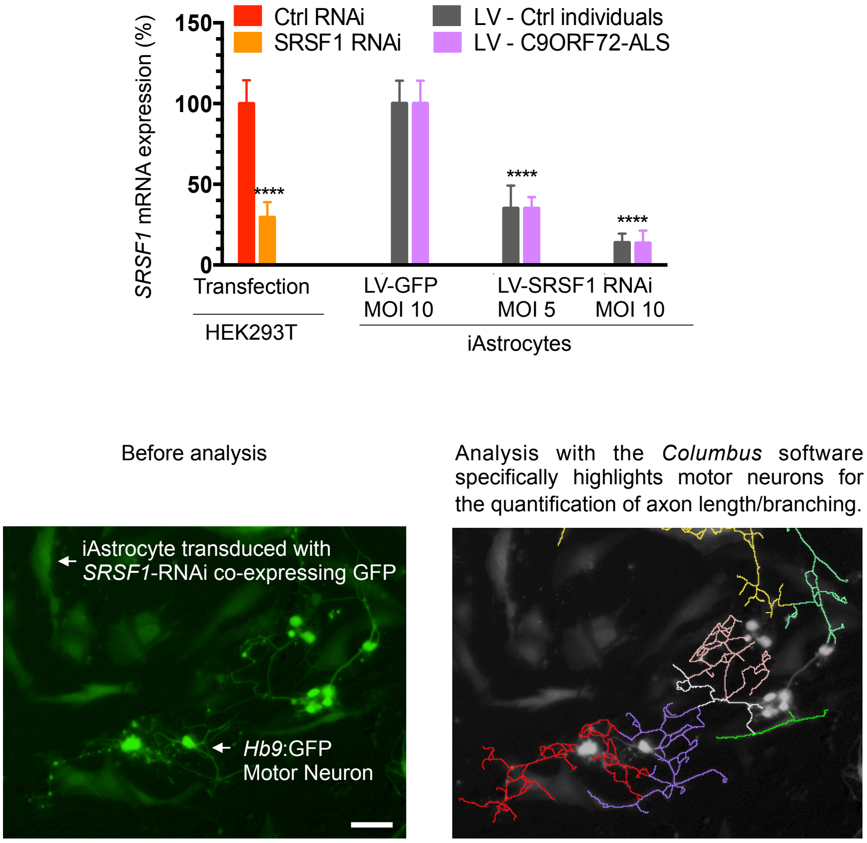

Supplementary Figure 2 | Co-cultures of mouse *HB9*:GFP motor neurons and *SRSF1*-RNAi transduced patient-derived astrocytes. (a) Lentiviral-mediated *SRSF1*-RNAi depletion was evaluated in HEK cells and iAstrocytes derived from control and C9ORF72-ALS patients. *SRSF1* transcript levels were quantified in transfected HEK cells and iAstrocytes transduced with increased MOI doses of LV-*SRSF1*-RNAi. *snRNA U1* transcript levels were used for normalization in three biological replicate experiments (mean ± SEM; two-way ANOVA with Tukey's correction for multiple comparisons; N (qPCR reactions) = 6). Statistical significance of data is indicated as follows: NS: non-significant, p≥0.05; *: p<0.05; **: p<0.01; ***: p<0.001; ****: p<0.0001. **(b)** High content imaging pictures showing how the Columbus analysis software recognizes *Hb9*:GFP motor neurons and the axons sprouting from them over *SRSF1*-RNAi-transduced astrocyte background. Scale bar: 50 µm.

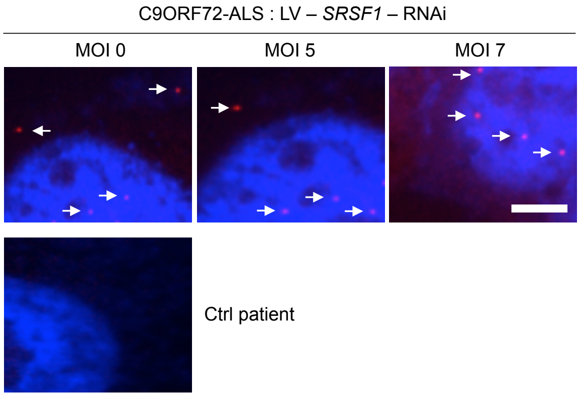

Supplementary Figure 3 | Depletion of SRSF1 leads to cytoplasmic reduction and nuclear accumulation of sense RNA foci. Representative images of sense RNA foci visualized using Cy3-CCCCGG Fluorescence In Situ Hybridization (red) by confocal microscopy in iAstrocytes trasnduced with increasing doses of *SRSF1*-RNAi (MOI 0, 5 and 7). The nuclei were stained in blue using DAPI. Arrows point to RNA foci. Cells with detectable RNA foci represent approximately 15-40% of the cell population depending on the individual patient-derived iAstrocyte line. Quantification was performed on 20-25 cells containing RNA foci (see Supplementary Table 1 for individual counts and Fig. 3e for bar chart). Scale bar: 3 μm.

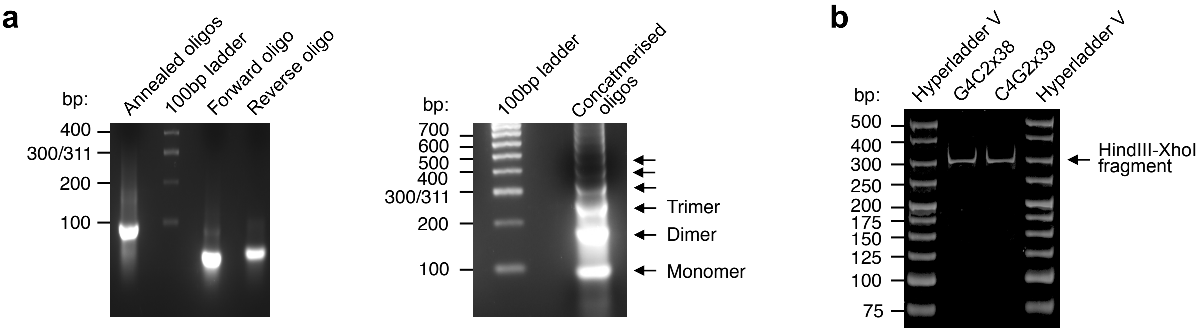

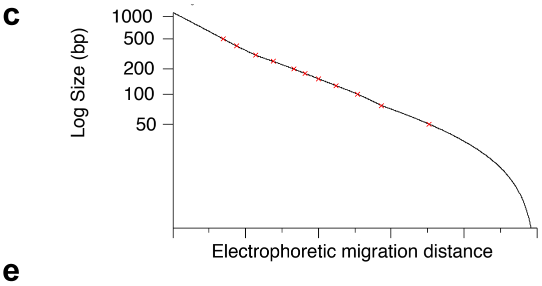

d

Construct	Theoretical Size	Calculated Size
G4C2x38	317 bp	315 bp
C4G2x39	316 bp	316 bp

e

RAN-G4C2x38 sense-repeats

...CTTATCGAAATTAATACGACTCACTATAGGGAGACCCAAGCTGGCTAGTTAAGCTTGGTACCGAGCTCGGATCCACTAGTCCAGTGTGGTGGAA
TTGGGGCCCGGGGCCGGGGCCGGGGCCGGGGCCGGGGCCGGGGCCGGGGCCGGGGCCGGGGCCGGGGCCGGGGCCGGGGCCGGGGCCGGGGCCGGGG
CCGGGGCCGGGGCCGGGGCCGGGGCCGGGGCCGGGGCCGGGGCCGGGGCCGGGGCCGGGGCCGGGGCCGGGGCCGGGGCCGGGGCCGGGGCCGGGGC
CGGGGCCGGGGCCGGGGCCGGGGCCGGGGCCGGGGCCGGGGCCGGGGAATTCTGCAGATATCCAGCACAGTGGCGGCCGCTCGAGTC**TAG**AGGGCCC
TTCGAACAAAAACTCATCTCAGAAGAGGATC**TGA**ATATGCATACCGGTCATCATCACCATCACCAT**TGA**GTT**TAA**ACCCGC**TGA**TCAGCCT*CGA*...

RAN-C4G2x39 antisense-repeats:

...CTTATCGAAATTAATACGACTCACTATAGGGAGACCCAAGCTGGCTAGTTAAGCTTGGTACCGAGCTCGGATCCACTAGTCCAGTGTGGTGGAA
TTCCCCGGCCCCGGCCCCGGCCCCGGCCCCGGCCCCGGCCCCGGCCCCGGCCCCGGCCCCGGCCCCGGCCCCGGCCCCGGCCCCGGCCCCGGCCCCG
GCCCCGGCCCCGGCCCCGGCCCCGGCCCCGGCCCCGGCCCCGGCCCCGGCCCCGGCCCCGGCCCCGGCCCCGGCCCCGGCCCCGGCCCCGGCCCCGG
CCCCGGCCCCGGCCCCGGCCCCGGCCCCGGCCCCGGCCCCGGCCCCAATTCTGCAGATATCCAGCACAGTGGCGGCCGCTCGAGTC**TAG**AGGGCCCT
TCGAACAAAAACTCATCTCAGAAGAGGATC**TGA**ATATGCATACCGGTCATCATCACCATCACCAT**TGA**GTT**TAA**ACCCGC**TGA**TCAGCCT*CGA*...

Supplementary Figure 4 | Generation of RAN-dependent uninterrupted G4C2-sense and C4G2-antisense repeat constructs. a, Agarose gels confirming both the annealing and concatemerisation of the G4C2x15 oligonucleotides. Arrows point to monomeric and multimeric forms of annealed oligonucleotides. **b,** Trimeric oligonucleotides were treated with Mung Bean nuclease for blunt cloning. The 8% acrylamide gel shows HindIII/XhoI inserts from pcDNA3.1/RAN constructs containing uninterrupted G4C2-sense and C4G2-antisense repeats with 5' and 3' flanking regions. **c,** Standard curve generated from the acrylamide gel analysis using the *Gene Tools Image* software. **d,** Table showing theroretical and experimental base pair size information for HindIII/XhoI inserts. The RAN constructs contain at least 38 G4C2-sense or 39 C4G2-antisense uninterrupted repeats based on the experimental size of the inserts (see sequences below). **e,** Sanger sequencing using betaine was also performed in the 5' and 3' directions using the T7 and T3 promoter sequencing primers respectively. Each sequence read covered the 5' or 3' flanking region and 9-16 G4C2 or C4G2 repeats prior to interruption. Sequencing traces are available on request. Boxes represent HindIII (AAGCTT) and XhoI (CTCGAG) cloning sites. The RNA transcripts generated from these constructs are highlighted in blue (flanking regions) and red (sense or antisense repeats). Sequences highlighted in black/underlined correspond to the 3'end of the promoter sequence and in black/italics to the start of the terminator sequence. Note the absence of initiating codons (ATG) in both sense and antisense transcripts generated from the RAN-dependent DPR-expression constructs. Stop codons are shown in all frames in bold. The repeat constructs expressing 15 repeats in sense or antisense orientation are identical except that they only contain 15 repeats. Sequencing and size analysis further showed that the number of repeats remained stable over multiple rounds of transformation and replication in NEB® 10-beta *E. coli* (*New England Biolabs*).

a

Expression of synthetic poly-Gly-Pro x36 DPRs independent of G4C2 repeats.

```
ATG GGC AAA CCG ATT CCG AAC CCG CTG CTG GGC CTG GAT AGC ACC CTC GAG AAT GAT CCC ACC ATG
GGC CCT GGC CCT GGA CCA GGA CCT GGC CCC GGA CCC GGT CCA GGT CCC GGC CCA GGC CCC GGT CCC
GGC CCT GGA CCA GGC CCA GGA CCA GGA CCA GGC CCA GGT CCC GGA CCA GGA CCC GGA CCT GGC CCA
GGC CCT GGC CCT GGC CCT GGC CCC GGA CCA GGC CCT GGA CCC GGC CCT GGT CCC GGC CCA GGA CCC
GGA CCA GGA CCT GGC CCT TAA
```

b

Expression of synthetic poly-Gly-Ala x36 DPRs independent of G4C2 repeats.

```
ATG GGC AAA CCG ATT CCG AAC CCG CTG CTG GGC CTG GAT AGC ACC CTC GAG AAT GAT CCC ACC ATG
GGA GCT GGT GCT GGT GCA GGC GCT GGC GCA GGG GCA GGC GCT GGT GCT GGG GCT GGT GCC GGG GCT
GGG GCA GGC GCA GGG GCT GGT GCC GGT GCA GGC GCA GGG GCT GGG GCT GGC GCT GGT GCC GGC GCA
GGC GCG GGT GCC GGC GCA GGG GCT GGT GCA GGG GCC GGT GCT GGC GCG GGT GCA GGG GCC GGT GCA
GGG GCA GGC GCA GGC GCT TAA
```

Supplementary Figure 5 | Generation of synthetic constructs expressing DPRs independently of RAN-translation and G4C2 repeat hexanucleotides. (a) Nucleotide sequence encoding poly-Gly-Pro x36 DPRs. **(b)** Nucleotide sequence encoding poly-Gly-Ala x36 DPRs. The DPR sequence is highligted in blue. The ATG start codon is highlighted in red while the TAA stop codon is highlighted in bold. A V5-tag is also present and highlighted in green.

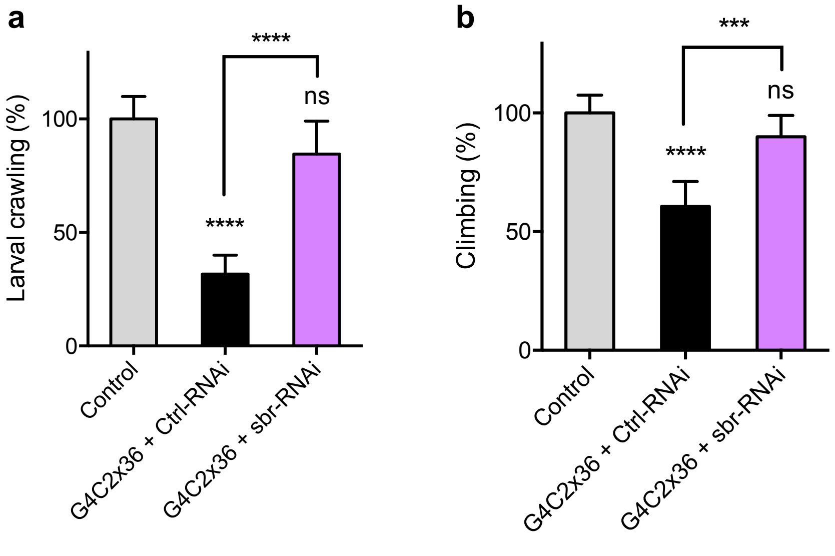

Supplementary Figure 6 | Partial loss of sbr/NXF1 restore locomotor deficits in G4C2x36 expressing flies. Neuronal expression of G4C2x36 causes larval crawling **(a)** and adult climbing **(b)** deficits that are both restored by *sbr* depletion (mean ± 95% CI normalized to Control; Kruskal-Wallis non-parametric test with Dunn's correction for multiple comparisons; N (larvae) = 10; N (adults) = Control (*GAL4/luciferase-RNAi*): 105, G4C2x36 + Ctrl-RNAi: 70, G4C2x36 + sbr-RNAi: 72). Statistical significance of data is indicated as follows: NS: non-significant, p≥0.05; *: p<0.05; **: p<0.01; ***: p<0.001; ****: p<0.0001.

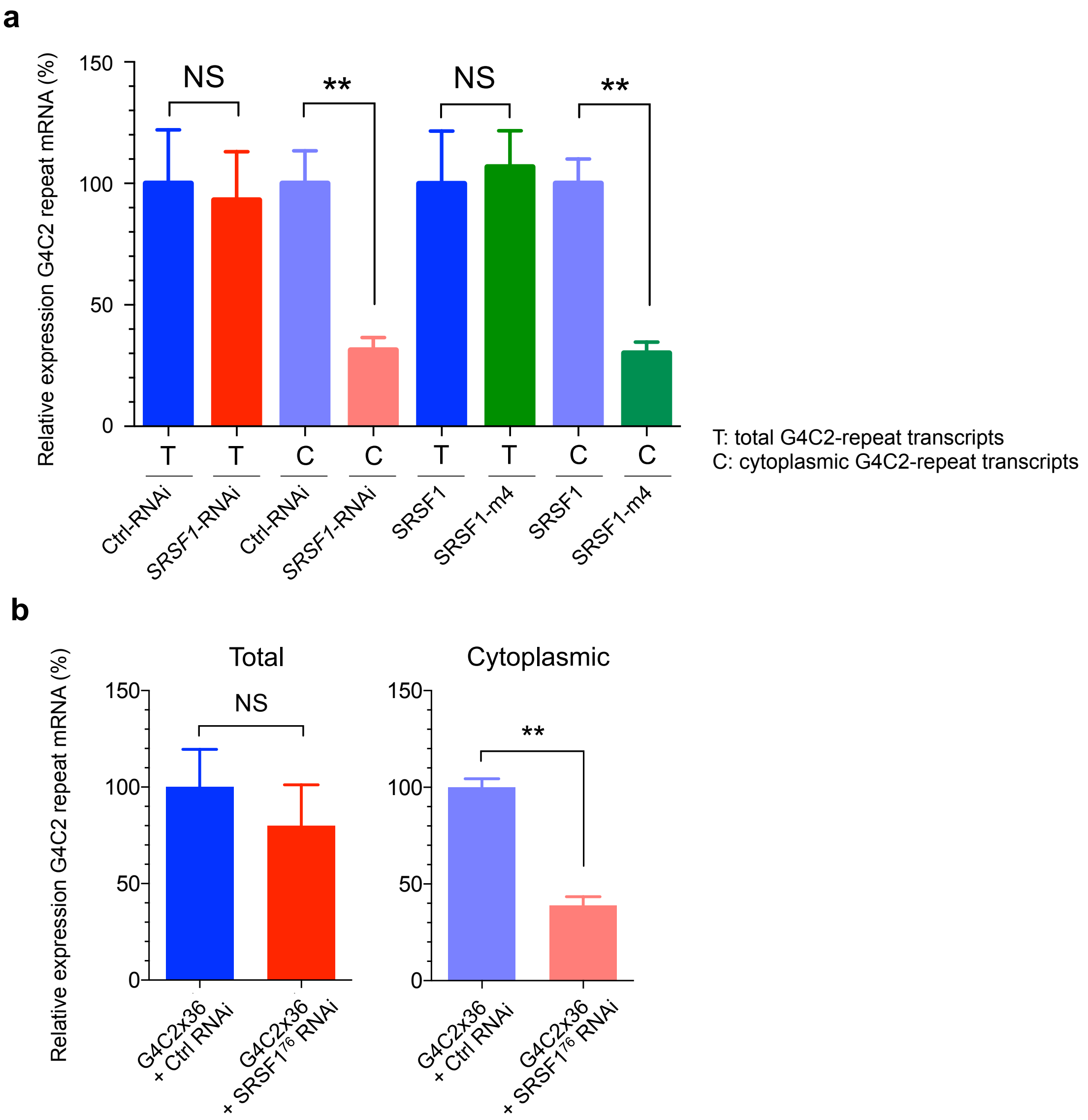

Supplementary Figure 7 | Depleting SRSF1 or inhibiting its sequestration and interaction with NXF1 alter the cytoplasmic levels of hexanucleotide repeat transcripts but not their total levels.
(a) N2A cells co-transfected with G4C2x38 and either Ctrl or *SRSF1*-RNAi plasmids (left part) and either FLAG-tagged SRSF1 aa11-196 wild type (SRSF1) or SRSF1-m4 (right part) were subjected to cellular fractionation using hypotonic lysis to yield cytoplasmic fractions (Fig. 6e). Total and cytoplasmic G4C2-repeat sense transcript levels were normalized to *U1* snRNA levels in three biological replicate experiments (mean ± SEM; one-way ANOVA with Tukey's correction for multiple comparisons; N (qRT-PCR reactions) = 6). **(b)** *Drosophila* expressing G4C2x36 and either control (Ctrl)-RNAi or *SRSF1*-RNAi. Whole flies were subjected to cellular fractionation using hypotonic lysis to yield cytoplasmic fractions (Fig. 6g). Total cytoplasmic G4C2-repeat sense transcript levels were normalized to *Tub84b* levels in three biological replicate experiments (mean ± SEM; paired *t*-test; N (qRT-PCR reactions) = 3). Statistical significance of data is indicated as follows: NS: non-significant, $p \geq 0.05$; *: $p<0.05$; **: $p<0.01$; ***: $p<0.001$; ****: $p<0.0001$.

RAN-G4C2x38 sense-repeats with 3x V5 tags

...CTTATCGAAATTAATACGACTCACTATAGGGAGACCCAAGCTGGCTAGTTAAGCTTGGTACCGAGCTCGGATCCACTAGTCCAGTGTGGTGGAA
TTGGGGCCCGGGGCCGGGGCCGGGGCCGGGGCCGGGGCCGGGGCCGGGGCCGGGGCCGGGGCCGGGGCCGGGGCCGGGGCCGGGGCCGGGGCCGGGG
CCGGGGCCGGGGCCGGGGCCGGGGCCGGGGCCGGGGCCGGGGCCGGGGCCGGGGCCGGGGCCGGGGCCGGGGCCGGGGCCGGGGCCGGGGCCGGGGC
CGGGGCCGGGGCCGGGGCCGGGGCCGGGGCCGGGGCCGGGGCCGGGGAATTCTGCAGATATCCAGCACAGTG GCGGCCGC TG GGCAAGCCCATC
CCCAACCCCCTGCTCGGTCTGGACAGCACCGGC**TAA** C GGCAAGCCCATCCCCAACCCCCTGCTCGGTCTGGACAGCACCGGC**TAA** C GGCAAGC
CCATCCCCAACCCCCTGCTCGGTCTGGACAGCACCGGC**TAA** CTCGAGTCTAGATC**TAG**AGGGCCCTTCGAACAAAAACTCATCTCAGAAGAGGATC
TGAATATGCATACCGGTCATCATCACCATCACCAT**TGA**GTT**TAA**ACCCGC**TGA**TCAGCCT *CGA*...

Supplementary Figure 8 | Transcript sequence . Boxes represent the HindIII (AAGCTT) and XhoI (CTCGAG) cloning sites used to clone the G4C2x38 annealed oligonucleotides (Supplementary Fig. 4). A synthetic construct encoding for the 3x V5 tags (sequences highlighted in orange, green and violet) with 3 stop codons (TAA, underlined/bold) were cloned in a second step using the NotI (GCGGCCGC) and XbaI (TCTAGA) sites. The RNA transcript generated from this construct is highlighted in blue (flanking regions) and red (38 G4C2-sense repeats) and orange, green and violet (3x V5 tags). Sequences highligted in black/underlined correspond to the 3'end of the promoter sequence and in black/italics to the start of the terminator sequence. Note the absence of initiating codons (ATG) in the transcript generated from the RAN-dependent DPR-expression construct. Sequencing and size analysis further showed that the number of repeats remained stable over multiple rounds of transformation and replication in NEB® 10-beta *E. coli (New England Biolabs*).

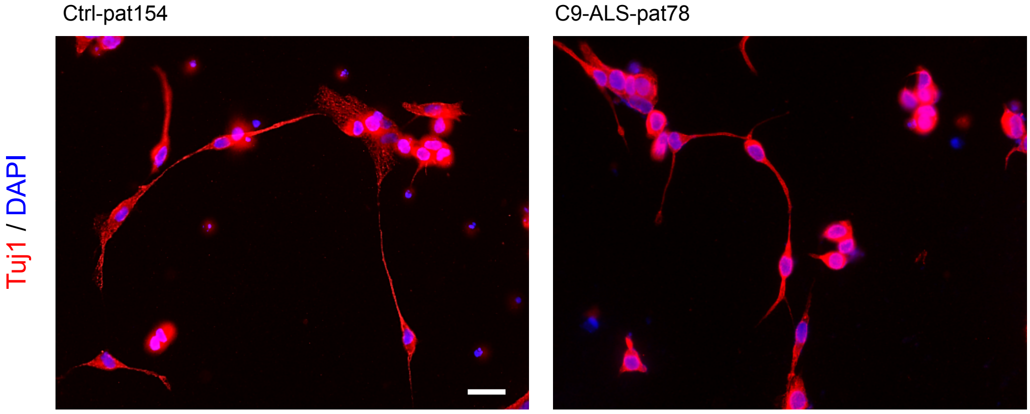

Supplementary Figure 9 | iNPC-differentiation of neurons derived from patient fibroblasts. Tuj1 immunofluorescence microscopy was performed on neurons differentiated from induced-Neural Progenitor Cells (iNPCs) derived from control (Ctrl-pat154) or C9ORF72-ALS (C9-ALS-pat78) patient fibroblasts using the red channel. DAPI was used to stain nuclei in blue. Scale bar: 50 μm.

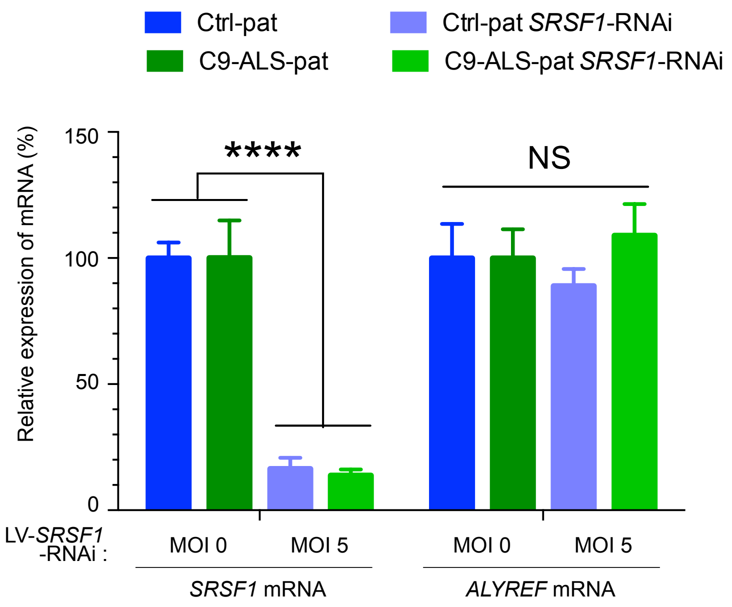

Supplementary Figure 10 | Evaluating the efficiency of lentiviral-mediated SRSF1-RNAi depletion in iNeurons derived from control and C9ORF72-ALS patients. *SRSF1* transcript levels were quantified in transfected HEK cells and iAstrocytes transduced with increased MOI doses of LV-*SRSF1*-RNAi. *snRNA U1* transcript levels were used for normalization in two comtrol (pat154, pat 155) or C9-ALS (pat78, pat183) cell lines in two technical replicates for each of two biological replicate experiments (mean ± SEM; two-way ANOVA with Tukey's correction for multiple comparisons; N (qPCR reactions) = 8). Statistical significance of data is indicated as follows: NS: non-significant, $p \geq 0.05$; *: $p<0.05$; **: $p<0.01$; ***: $p<0.001$; ****: $p<0.0001$.

Figure 3a

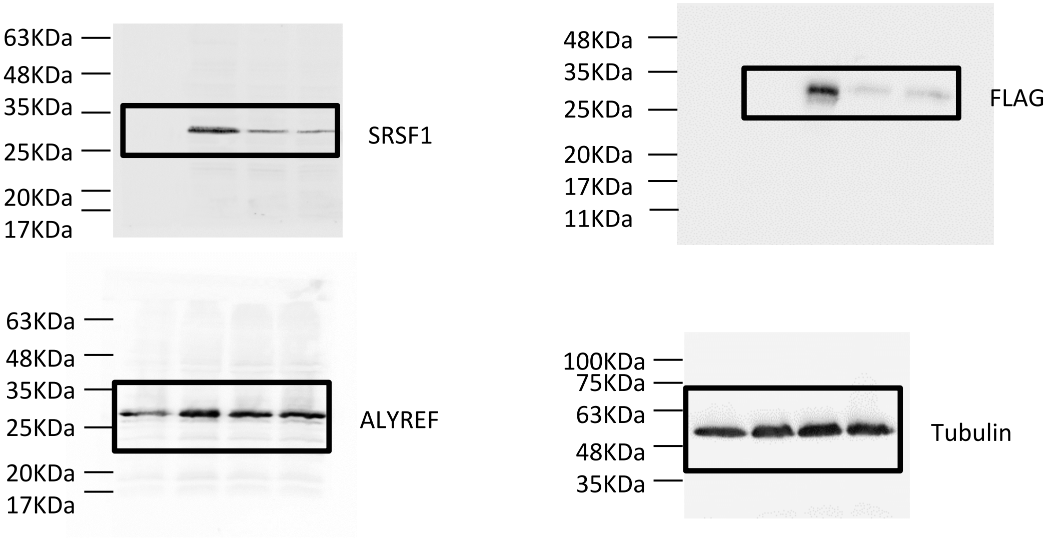

Figure 4c

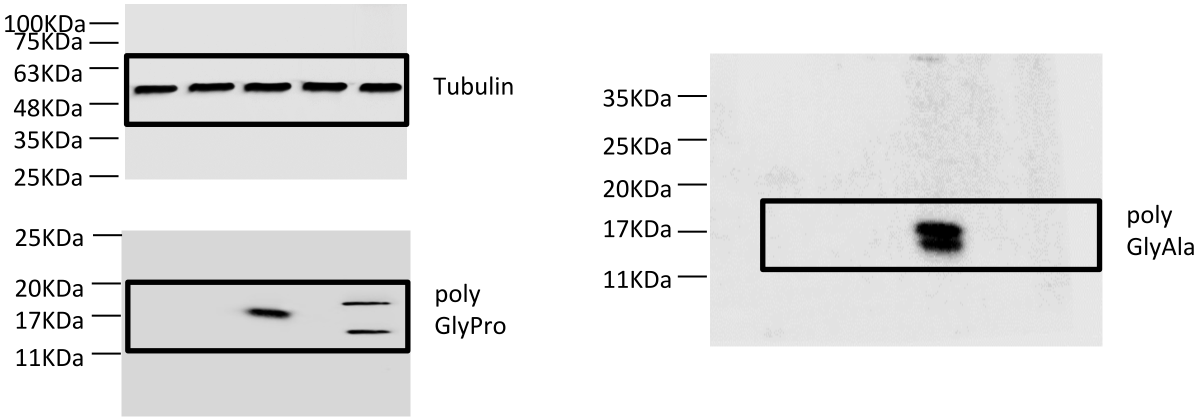

Supplementary Figure 11 | Uncropped western blot images for figures 3a and 4c. Molecular weight (kDa) of the pre-stained protein ladder bands are indicated on the left side of the panels. Antibodies used to probe the nitrocellulose membranes are indicated on the right side. Rectangles represent the cropped images shown in figures.

Figure 5a

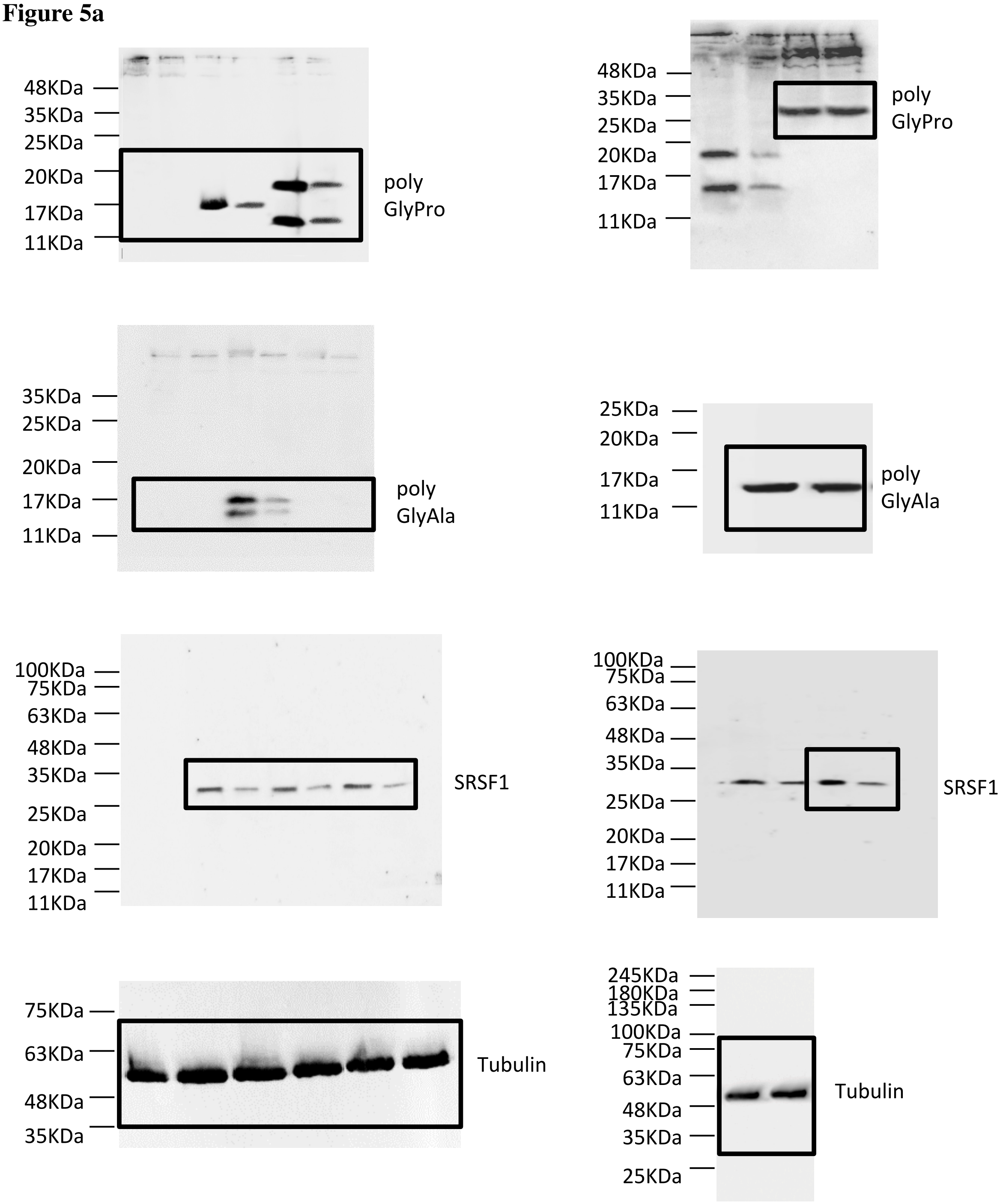

Supplementary Figure 12 | Uncropped western blot images for figure 5a. Molecular weight (kDa) of the pre-stained protein ladder bands are indicated on the left side of the panels. Antibodies used to probe the nitrocellulose membranes are indicated on the right side. Rectangles represent the cropped images shown in figures.

Figure 5e

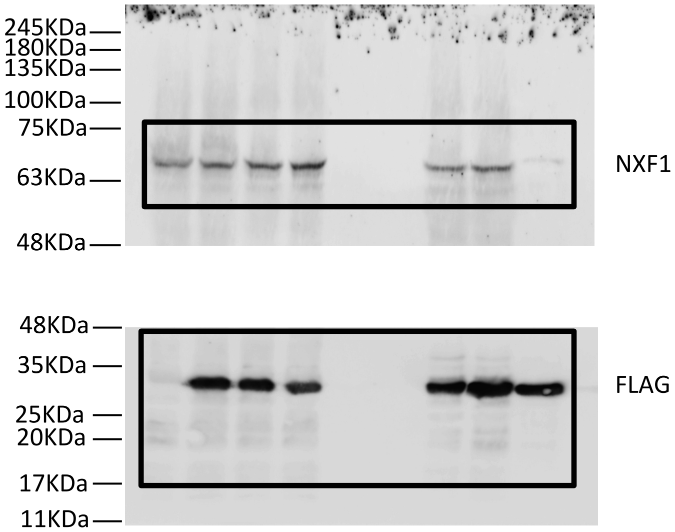

Supplementary Figure 13 | Uncropped western blot images for figure 5e. Molecular weight (kDa) of the pre-stained protein ladder bands are indicated on the left side of the panels. Antibodies used to probe the nitrocellulose membranes are indicated on the right side. Rectangles represent the cropped images shown in figures.

Figure 5f

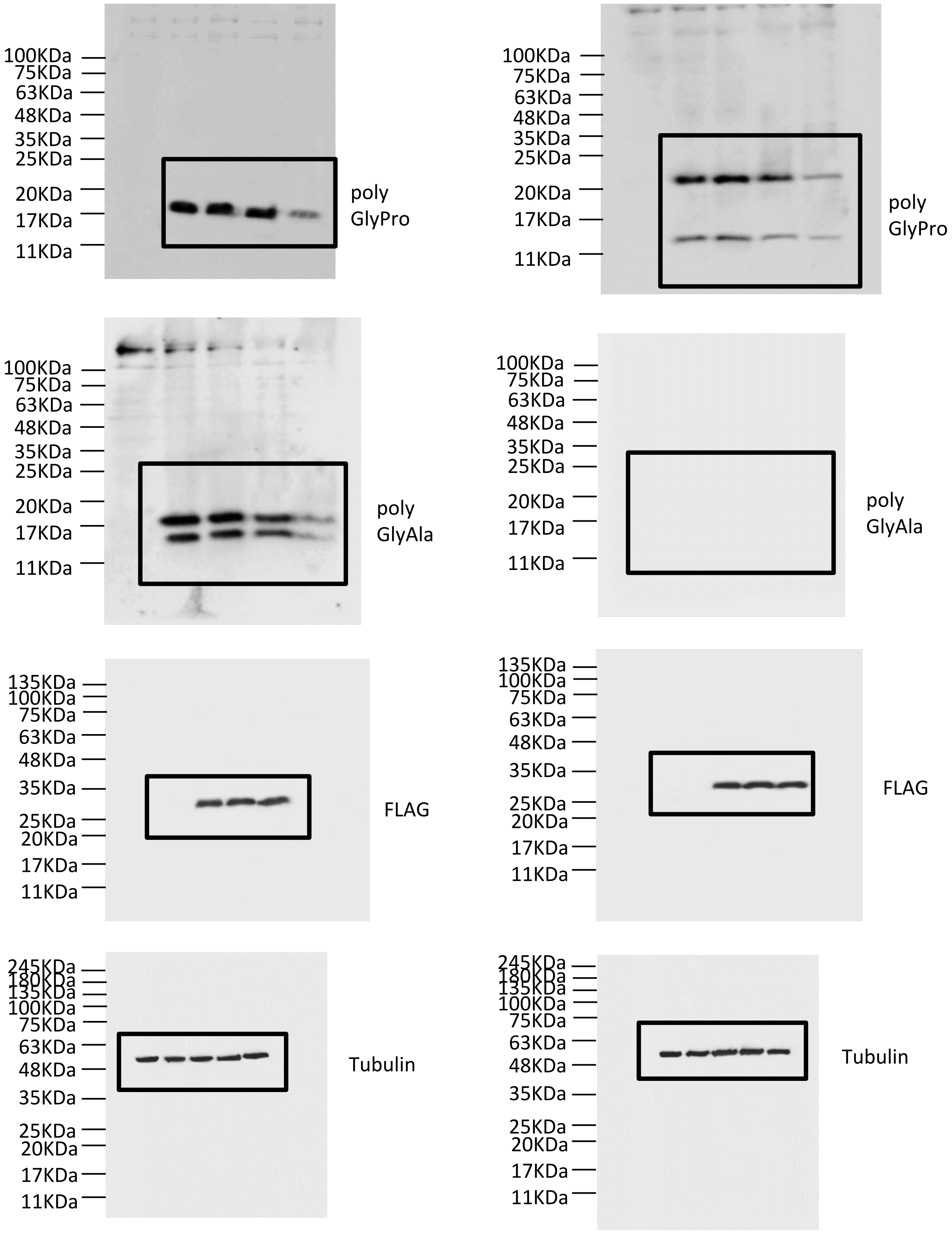

Supplementary Figure 14 | Uncropped western blot images for figure 5f. Molecular weight (kDa) of the pre-stained protein ladder bands are indicated on the left side of the panels. Antibodies used to probe the nitrocellulose membranes are indicated on the right side. Rectangles represent the cropped images shown in figures.

Figure 6e

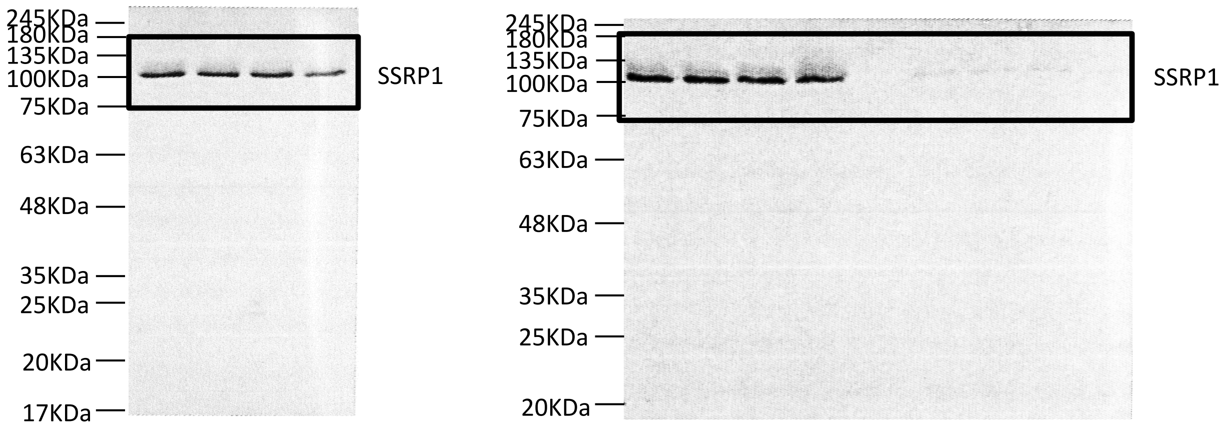

Supplementary Figure 15 | Uncropped western blot images for figure 6e. Molecular weight (kDa) of the pre-stained protein ladder bands are indicated on the left side of the panels. Antibodies used to probe the nitrocellulose membranes are indicated on the right side. Rectangles represent the cropped images shown in figures.

Figure 6g

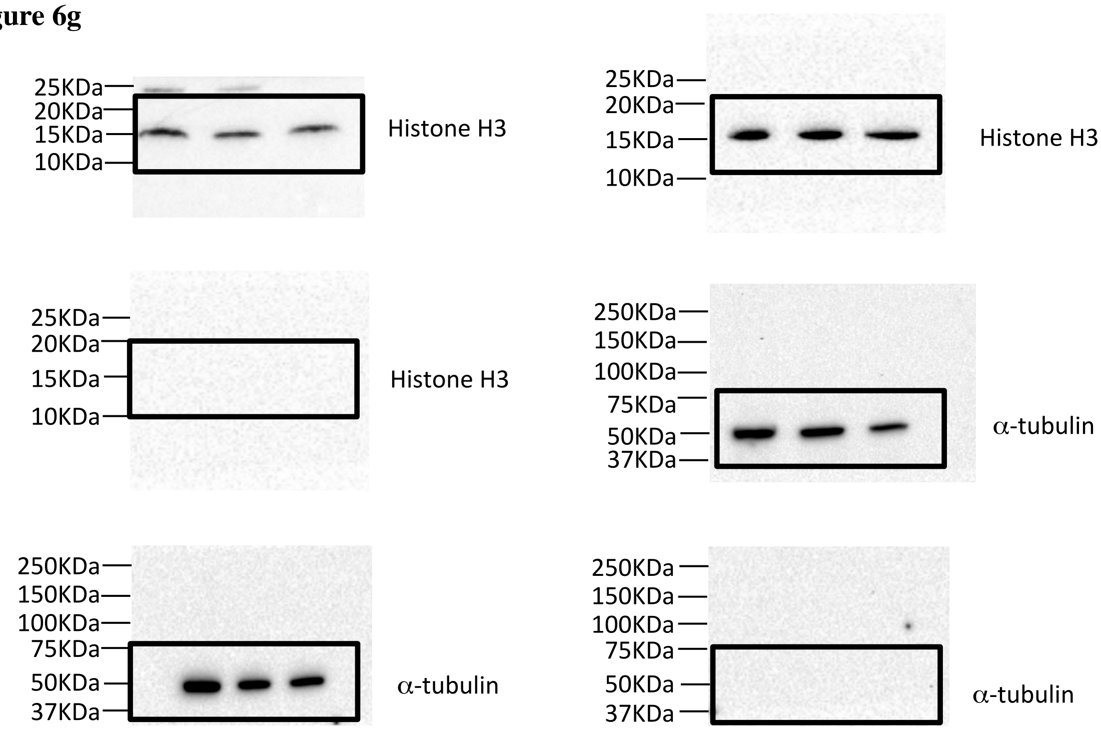

Figure 8d

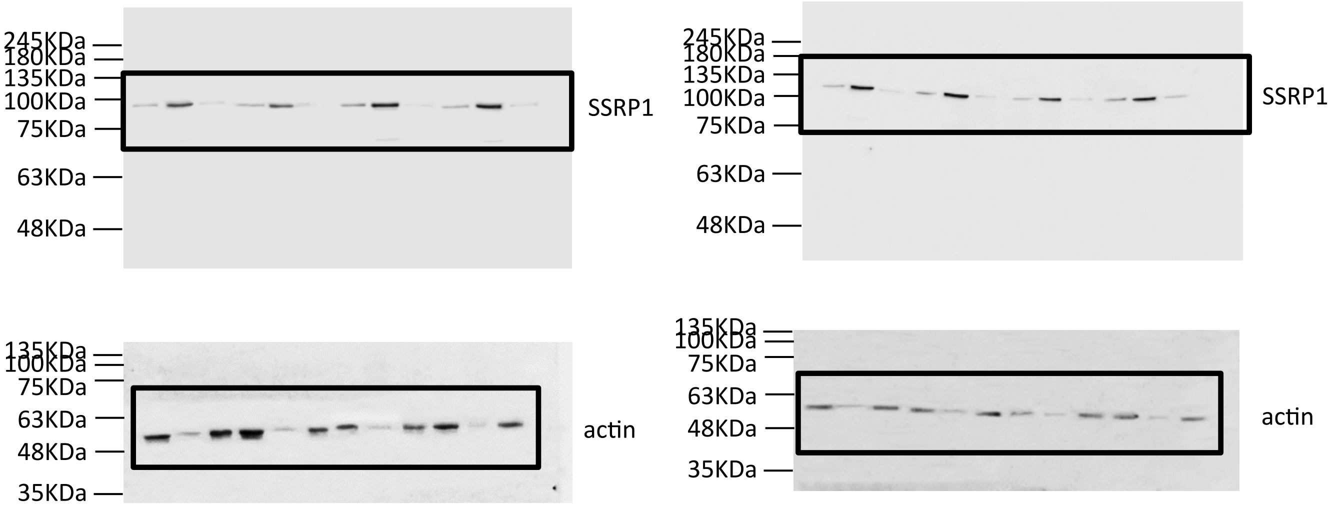

Supplementary Figure 16 | Uncropped western blot images for figure 6g. Molecular weight (kDa) of the pre-stained protein ladder bands are indicated on the left side of the panels. Antibodies used to probe the nitrocellulose membranes are indicated on the right side. Rectangles represent the cropped images shown in figures.

Supplementary Table 1 | Number and cellular distribution of G4C2 RNA foci in iNPCs-derived astrocytes treated with increasing MOI of LV-*SRSF1*-RNAi

	iAstrocytes C9ORF72-ALS Patient 78						iAstrocytes C9ORF72-ALS Patient 183						iAstrocytes C9ORF72-ALS Patient 201					
SRSF1-RNAi MOI	0		5		7		0		5		7		0		5		7	
Foci	Nuc	Cyto	Nuc	Cyto	Nuc	Cyto	Nuc	Cyto	Nuc	Cyto	Nuc	Cyto	Nuc	Cyto	Nuc	Nuc	Nuc	Cyto
Raw counts	1	0	0	1	2	1	0	1	1	0	1	0	1	0	1	0	4	1
	2	0	1	0	1	0	2	0	1	0	1	0	2	0	1	0	2	1
	1	0	2	0	1	0	1	0	1	0	1	0	1	0	2	0	4	0
	1	1	0	2	2	0	1	0	0	1	2	1	1	0	1	0	1	1
	1	0	1	0	1	0	1	1	1	0	1	1	1	1	3	1	3	0
	0	2	1	0	2	1	0	1	3	0	1	0	3	1	1	0	1	0
	0	1	1	0	3	0	1	1	2	0	1	0	2	0	2	0	2	0
	0	1	1	0	2	0	1	1	3	0	1	0	0	1	2	0	1	0
	0	1	2	0	1	0	2	0	1	2	1	0	1	0	1	0	3	0
	1	0	1	0	1	0	0	1	0	1	3	0	1	0	1	1	3	0
	1	1	2	0	4	1	0	1	1	0	1	0	1	1	1	0	2	0
	1	1	1	0	1	0	1	0	1	0	1	0	1	0	1	1	3	0
	2	0	3	1	2	0	1	0	1	0	5	1	0	2	2	0	1	0
	1	2	2	0	1	0	0	1	1	2	4	1	1	0	1	0	2	0
	0	1	1	0	3	0	2	1	0	1	2	0	1	2	2	1	1	0
	1	0	1	1	4	1	1	2	1	1	1	0	0	1	1	0	3	1
	1	0	1	0	1	0	1	0	1	1	3	0	1	0	0	1	1	0
	0	2	0	1	1	0	1	1	1	0	1	0	1	0	2	0	1	0
	2	1	1	0	2	0	0	1	1	0	3	1	1	0	1	1	2	0
	1	0	2	1	1	0	1	1	1	0	2	0	0	1	1	1	2	0
	1	1			2	0	3	1	0	1	0	1	0	1	1	0	1	0
					2	1			1	0	1	0	1	1	2	0	0	1
									0	1			0	1	4	1		
									3	1			0	1				
Average	0.857	0.714	1.200	0.350	1.818	0.227	0.952	0.714	1.083	0.500	1.682	0.273	0.875	0.583	1.478	0.348	1.955	0.227
S.E.M.	0.143	0.156	0.172	0.131	0.204	0.091	0.176	0.122	0.180	0.135	0.258	0.097	0.151	0.133	0.176	0.102	0.232	0.091

Nuc = Nuclear; Cyto = Cytoplasmic

Supplementary Note 1 | References and sequences of insertions for the *Drosophila* RNAi lines

SRSF1 (SF2/ASF) - RNAi lines:
v27775: FlyBase ID = FBst0457117
v27776: FlyBase ID = FBst0457118
Independent insertion lines, both lines carry the following inverted repeat sequence:
ATGCCGACGA TGCGGTGAAG GCGCGCGACG GCTACGACTA CGATGGGTAT
CGTCTGCGCG TGGAGTTCCC GCGGGGCGGT GGTCCTGGAA GCTACCGCGG
CGGCAACCGC AATGACCGAA GCCGCGACGG TGGGGGACGG ATGGGCGGAC
GCGGACCGCC AGCCAAGCGC TCGCAGTACC GCGTCATGGT TACTGGACTG
CCCGCCTCCG GATCGTGGCA AGATCTCAAG GATCACATGC GCGAGGCCGG
CGACGTCTGC TTCGCGGACA CTTACAAGGA TGGTTCCGGC GTCGTTGAGT
TCCTGCGCCA CGAGGACATG AAGTACGCAA TCAAAAAATT GGACGACTCT
CGCTTCCGA

ALYREF (Ref1) - RNAi lines:
v12301 (GD): FlyBase ID = FBst0450381 - the line carry the following inverted repeat sequence:
GGTCCGATAA AGAAGGCGGC AGTGCACTAC GATCGCTCCG GTCGCTCGTT
GGGCACCGCT GACGTGATTT TCGAACGTCG CGCCGACGCC TTGAAGGCCA
TTAAACAGTA CCATGGCGTA CCTTTGGACG GACGCCCTAT GACCATTCAG
CTGGCCGTCT CAGACGTGGC CGTGTTGACC CGTCCCGTAG CCGCCACCGA
TGTCAAGCGT CGCGTGGGTG GTACTGCACC AACTTCATTC AAGCGTGGTG
GTGGCCAAGC TGGTGGCACG GCGCGTCGCG GCTTCAAACG TCCGGTCGGT
GGCAAGCCGG CGGCAGGCGG CCAGCGACGG GAGCGCAAGG CCCCGCCCAC
TGCTGAGGAG CTGGACGCCG AACTGGACTC A

v104471 (KK): FlyBase ID = FBst0476329 - - the line carry the following inverted repeat sequence:
GTCGAACTTG ATAAAGCGCA TTTCTAAATA CAATAAATAC AGCATCAAAT
GTATTTCAGT TATCTTAACA TCCGCCGCAT TGGCAAAACT AACAATTAAT
GGATAAATGC GCAAGTGGTT GATTGATTTG ATGTCCGATG CTTTCAAAGA
TCTGCTCCTG GGCGCGGCGT TGTCGATGCG TTTGCATTTA TGTACCATGC
GGGGGGTGTC CATATGGTAG GCTTAAAACT ATAGATTGGG CTGCTCTTCT
ATTCTTGTTA GACTAATTCA GACTATTCAC TATTTAGATC TTCATGTCGT
TGATGTATGA GTCCAGTTCG GCGT

Supplementary Note 2 | Sequences of designed oligonucleotides and miRNA hairpins

miRNA hairpins were designed against human *SRSF1* (NCBI Reference Sequence: NM_006924.4, mRNA), mouse *SRSF1* (NCBI Reference Sequence: NM_173374.4, mRNA) and rat *SRSF1* (NCBI Reference Sequence: NM_001109552.2, mRNA). The *SRSF1* sequence targeted by miRNA hairpin 1 is identical in human, mouse and rat *SRSF1*. The blue regions highlighted in sequences below represent the mature miR RNAi sequences which targets the complementary sense sequences on *SRSF1* (highlighted in red):

1/ Targeted human, mouse and rat *SRSF1* miR1 sequence (TTAAAGTTGATGGGCCCAGAA) respectively starts at 784 nt (NCBI RefSeq NM_006924.4 - RRM2 region), 1,041 nt (NCBI RefSeq NM_173374.4 - RRM2 region) and 699 nt (NCBI RefSeq: NM_001109552.2 - RRM2 region):
- human/mouse/rat *SRSF1* - miR1 -Top strand:
5'- TGCTGTTCTGGGCCCATCAACTTTAAGTTTTGGCCACTGACTGACTTAAAG TTTGGGCCCAGAA -3'
- human/mouse/rat *SRSF1* - miR1 - Bottom strand:
5'- CCTGTTCTGGGCCCAAACTTTAAGTCAGTCAGTGGCCAAAACTTAAAGTTG ATGGGCCCAGAAC -3'

2/ Targeted human SRSF1 miR2 sequence (AATGGTATGACTCCAAGTGCT) starts at 1436 nt (NCBI RefSeq NM_006924.4 - 3'UTR region):
- human *SRSF1* - miR2 - Top strand:
5'- TGCTGAGCACTTGGAGTCATACCATTGTTTTGGCCACTGACTGACAATGGT ATCTCCAAGTGCT -3'
- human *SRSF1* - miR2 - Bottom strand:
5'- CCTGAGCACTTGGAGATACCATTGTCAGTCAGTGGCCAAAACAATGGTAT GACTCCAAGTGCTC -3'

3/ Targeted mouse *SRSF1* miR2 sequence (AATGTCTATTCTGCTCTGGTT) starts at 1,473 nt (NCBI RefSeq NM_173374.4 - 3'UTR region):
- mouse *SRSF1* – miR2 - Top strand:
5'- TGCTGAACCAGAGCAGAATAGACATTGTTTTGGCCACTGACTGACAATGT CTACTGCTCTGGTT -3'
- mouse *SRSF1* - miR2 - Bottom strand:
5'- CCTGAACCAGAGCAGTAGACATTGTCAGTCAGTGGCCAAAACAATGTCTA TTCTGCTCTGGTTC -3'

Supplementary Note 3 | Sequences of qPCR primers used in the study

Drosophila SRSF1 (designed using Primer-BLAST)
Fwd: 5'-TACCGCGTCATGGTTACTGG-3'
Rev: 5'-GTACGCGAATGTAGGCAACC-3'

Drosophila ALYREF (designed using Primer-BLAST)
Fwd: 5'- CGATATGTACGACGGACCGAA-3'
Rev: 5'- CGGACCAAAGTCGTTGAAGAG-3'

Drosophila Tub84b (described in reference[68])
Fwd: 5'-TGGGCCCGTCTGGACCACAA-3'
Rev: 5'-TCGCCGTCACCGGAGTCCAT-3'

Drosophila C9 3'UTR (described in reference[69])
Fwd: 5'-TTCCAACCTATGGAACTGATGA-3'
Rev: 5'-GGTTTTCCTCATTAAAGGCATTC-3'

Human *SRSF1* (designed using Primer-BLAST)
Fwd: 5'-CCGCATCTACGTGGGTAACT-3'
Rev: 5'-TCGAACTCAACGAAGGCGAA-3'

Human *ALYREF* (designed using Primer-BLAST)
Fwd: 5'-TCTGGTCGCAGCTTAGGAAC-3'
Rev: 5'-CCACCTCTGTTTACGCTCTGT-3'

Human *U1* snRNA (designed using Primer-BLAST)
Fwd: 5'-CCATGATCACGAAGGTGGTT-3'
Rev: 5'-ATGCAGTCGAGTTTCCCACA-3'

Human *SMN* (described in reference[70])
Fwd 5'-CTTGTGAAACAAAATGCTTTTTAACATCCAT-3'
Rev 5'-GAATGTGAGCACCTTCCTTCTTTTT-3'

Human *JUN* (designed using Primer BLAST)
Fwd 5'-GAACTGCACABCCAGAACAC-3'
Rev 5'TGGGTTGAAGTTGCTGAGG-3'

C9RAN (designed using Primer-BLAST). Primers anneal downstream of the G4C2 or C4G2 repeat sequences in the 3'UTR of mRNA transcribed from pcDNA3.1 constructs.
Fwd 5'-GGGCCCTTCGAACCCCCGTC-3'
Rev: 5'GGGAGGGGCAAACAACAGAT-3'

Human *C9ORF72* Exon-1 Forward (designed using Primer BLAST)
5'-TCAAACAGCGACAAGTTCCG-3'

Human *C9ORF72* Exon-3 Reverse (designed using Primer BLAST)
5'-GTCGACATGACTGCATTCCA-3'

Human *C9ORF72* Intron-1 Reverse (designed using Primer BLAST)
5'-GGAGAGAGGGTGGGAAAAAC-3'
